# Estimation of vegetation water content using hyperspectral vegetation indices: a comparison of crop water indicators in response to water stress treatments for summer maize

**DOI:** 10.1186/s12898-019-0233-0

**Published:** 2019-04-29

**Authors:** F. Zhang, G. Zhou

**Affiliations:** 1 Chinese Academy of Meteorological Sciences, Beijing, 100081 China; 2 State Key Laboratory of Vegetation and Environmental Change, Institute of Botany, Chinese Academy of Sciences, Beijing, 100093 China

**Keywords:** Canopy water content, Hyperspectral remote sensing, Leaf equivalent water thickness, Live fuel moisture content, Summer maize

## Abstract

**Background:**

Vegetation water content is one of the important biophysical features of vegetation health, and its remote estimation can be utilized to real-timely monitor vegetation water stress. Here, we compared the responses of canopy water content (CWC), leaf equivalent water thickness (EWT), and live fuel moisture content (LFMC) to different water treatments and their estimations using spectral vegetation indices (VIs) based on water stress experiments for summer maize during three consecutive growing seasons 2013–2015 in North Plain China.

**Results:**

Results showed that CWC was sensitive to different water treatments and exhibited an obvious single-peak seasonal variation. EWT and LFMC were less sensitive to water variation and EWT stayed relatively stable while LFMC showed a decreasing trend. Among ten hyperspectral VIs, green chlorophyll index (CI_green_), red edge normalized ratio (NR_red edge_), and red-edge chlorophyll index (CI_red edge_) were the most sensitive VIs responding to water variation, and they were optimal VIs in the prediction of CWC and EWT.

**Conclusions:**

Compared to EWT and LFMC, CWC obtained the best predictive power of crop water status using VIs. This study demonstrated that CWC was an optimal indicator to monitor maize water stress using optical hyperspectral remote sensing techniques.

## Background

Drought is one of the most important impacts of global climate change on terrestrial ecosystems. It is also a major environmental abiotic stress factor that currently reduces crop yield worldwide [1]. Among many natural hazards, the effects of drought on the world’s agricultural production is most prominent and its influences are the sum of other natural hazards [2]. Agricultural drought mainly reflects soil water status as well as crop growth and morphology, which can be used to reflect the degree of soil water deficit to crop water demand. When a crop is in a water deficit status, water stress will act directly on crop growth and development, photosynthesis, dry mass production, and seed production, and ultimately, water stress will lead to crop production reduction [3]. Therefore, how to accurately assess and monitor crop water stress is not only the key for adopting scientific countermeasures to reduce adverse effects, but also essential research for monitoring, warning, and assessing agricultural drought.

In recent years, remote sensing techniques have been widely utilized to monitor and assess crop drought, forest and grass fire danger, land-use change, and crop production [4]. Usually, studies on remote monitoring and assessment by field spectroscopy techniques mainly involve crop leaf structural and biochemical component information such as chlorophyll, nitrogen content, dry mass content, and water content [5–16]. Among them, remote estimation of vegetation water content can provide important implications on vegetation physiological status detection [7, 12, 17–20], agricultural irrigation decision [10, 12, 13], and drought assessment [21, 22]. Remote sensing techniques can be used to effectively monitor and diagnose vegetation water conditions, accurately reflect physiological status of vegetation under water stress, rapidly recognize drought, and immediately adopt irrigation measures [10, 12, 13, 22, 23].

The commonly used physiological indicators to assess plant water conditions mainly include stomatal conductance [3], leaf water potential [3, 24], canopy water content (CWC) [25–28], leaf equivalent water thickness (EWT) [25, 29], live fuel moisture content (LFMC) [11, 13, 30], and relative water content (RWC) [31–33]. Leaf water status has been widely used as an indicator of crop water stress [24, 34]. LFMC, the ratio of water mass to dry mass contained in live plant material, is not only affected by leaf moisture status, but also impacted by seasonal variation of dry mass [35]. LFMC represents the magnitude of fuel and is an important fuel property for determining fire danger and modeling fire behavior [36]. RWC, the ratio of leaf water content at the time of measurement to leaf water content at a swelling pressure level, is commonly used to assess the water status of plants and has been estimated using spectral data [36]. EWT, defined as quantity of water per unit leaf area, is more relevant to the water absorption of incoming radiation. Additionally, EWT plays a crucial role in biogeochemical processes such as photosynthesis, evaporation, and primary productivity [37, 38]. Its rapid decreases or shortage is an important early stress indicator [39]. CWC, expressed as the quantity of water per unit area of ground surface, is widely utilized to monitor vegetation water conditions [27, 28] and is determined not only by vegetation water status but also by crop growth and development stages [15]. Multi-scale and real-time monitoring of vegetation water status or crop water stress using remote sensing techniques has been conducted. However, up to now, there is still disagreement on which is the most suitable method among water content indicators for remotely monitoring crop water stress.

Maize is one of the most important crops in China and summer maize is a major food crop in North Plain China. This region is dominated by two main climatic properties, which are frequent drought and uneven distribution of inter-annual rainfall during the growing season [40]. Studies on the effects of drought on summer maize have been a subject of scientific interest in recent years [15]. However, thorough understanding of sensitive water indicators of summer maize in response to water stress and monitoring of maize water stress using hyperspectral remote sensing is still lacking. Although a number of studies have been conducted to construct empirical models using spectral vegetation indices (VIs), only a few study, e.g. Cao et al. [23], exploited a comprehensive dataset including not only extreme drought values but also extreme moist values from laboratory experiments.

Therefore, in this study, we collected canopy spectral reflectance from field spectrometry and its corresponding biological and environmental observation datasets based on water stress experiments of summer maize from 2013 to 2015. Then, we compared the responses of CWC, EWT, and LFMC to water stress and their estimations using spectral VIs. The objectives were to: (i) explore the differences of CWC, EWT, and LFMC in response to water stress treatments and their seasonal variations, (ii) clarify the effects of water stress treatments on canopy spectral VIs, and (iii) compare the predictions of CWC, EWT, and LFMC using spectral VIs. This study will provide important information for large-area, non-destructive, real-time monitoring and assessment of vegetation growth, crop drought, and crop production using optical remote sensing techniques.

## Methods

### Study area

This study was conducted at the Gucheng Ecological and Agricultural Research Station (39°08′N, 115°40′E, 15.2 m a.s.l.), Chinese Academy of Meteorological Sciences, in Dingxing county, Hebei province, China. This region belongs to a warm temperate continental monsoon climate zone with a mean annual air temperature and precipitation of 11.7 °C and 551.5 mm, respectively. The selected crop type was maize hybrid Zheng Dan 958, sown in late June and harvested in early October. A large water controlled experimental field was employed in which 2 × 4 experimental plots with natural field soil were used to plant maize. The rain-out shelters were movable, and the experimental plots except for control plots were sheltered when it rained. Thus, the rainfall was excluded by large electric rain-out shelters so that the water supply could be artificially controlled by standard irrigation [15].

### Experimental design and treatments

Responses of summer maize to water stress treatments were continuously monitored from 2013 to 2015 (Table 1). The study consisted of seven water treatments from 1 to 7 with different water irrigation regimes (120, 100, 80, 60, 40, 25, and 15 mm) and the corresponding rainfed control plots with no irrigation and a rainfed field control in 2013. Seven water treatments from 1 to 7 with water irrigation regimes (225, 150, 120, 90, 60, 30, and 10 mm) and the corresponding rainfed control plots with no irrigation were designed in 2014. In 2013, irrigation treatment was completed on 24 July at the seven-leaf stage of maize, while irrigation treatment was finished at the seedling stage in 2014. Additionally, only treatments 1 and 2 in 2013 were re-wetted with water amounts of 80 mm and 40 mm on 26 August at the flowering stage. In 2015, five water treatments from 1 to 5 were conducted with each treatment simulating different water stress gradients including one adequate water supply treatment 1, two continuous water stress treatments (slightly continuous water stress treatment 2 and moderately continuous water stress treatment 3); and two stable water stress treatments (slightly stable water stress treatment 4 and seriously stable water stress treatment 5) (Table 1). Except for four replicates for treatment 1 in 2015, three replicates were performed during the experiments with each plot being one replicate from 2013 to 2015.

**Table 1:** Experimental design and irrigation amounts (mm) in 2013–2015

Year	2013	2014	2015
Control plots	0 (natural rainfall) × 3 replicates	0 (natural rainfall) × 3 replicates	–
Treatment 1	120 + 80 × 3 replicates	225 × 3 replicates	Adequate water supply (RSWC: 75% ± 5%) × 4 replicates
Treatment 2	100 + 40 × 3 replicates	150 × 3 replicates	Slightly continuous water stress (RSWC: 75% ± 5% before the jointing stage + 16 mm) × 3 replicates
Treatment 3	80 × 3 replicates	120 × 3 replicates	Moderately continuous water stress (RSWC: 75% ± 5% before the jointing stage + 0 mm) × 3 replicates
Treatment 4	60 × 3 replicates	90 × 3 replicates	Slightly stable water stress (RSWC: 55% ± 5%) × 3 replicates
Treatment 5	40 × 3 replicates	60 × 3 replicates	Seriously stable water stress (RSWC: 35% ± 5%) × 3 replicates
Treatment 6	25 × 3 replicates	30 × 3 replicates	–
Treatment 7	15 × 3 replicates	10 × 3 replicates	–
Field control	0 (natural rainfall) × 4 replicates	–	–

“–” shows no experimental design or no treatment, “+” indicates rewetting irrigation and RSWC means relative soil water content

### Field measurements

A total of 26 sets of field reflectance spectra measurements were conducted on a nearly weekly basis between July to October as follows: 23 and 29 July, 8, 18, and 25 August, 5 and 20 September, 8 October in 2013; 10 and 18 July, 1, 7, and 19 August, 3, 16, and 27 September in 2014; 7 and 25 July, 4, 15, and 27 August, 2, 7, 15, and 24 September, and 9 October in 2015. An ASD FieldSpec3 spectroradiometer (Analytical Spectral Devices, Boulder, CO, USA) was used to measure spectral reflectance. The wavelength range was 350–2500 nm with a sampling interval of 1.4 nm below 1000 nm and 2 nm above 1000 nm. The spectral resolution was 3 nm and 10 nm in the 350–1000 nm and 1000–2500 nm ranges, respectively. Spectral measurements were made on days with clear skies between 11 h and 14 h. The fiber optics, with a field of view of 25°, were handheld approximately 1–1.3 m above the undisturbed maize canopy at the nadir position at each treatment plots and field control for every observation. In addition, 20 spectral readings were taken for each spectral measurement above the maize canopy per experimental plot. The mean value of spectral reflectance averaged over these 20 spectral measurements was used as the spectral reflectance of each experimental plot. During spectral measurements, a standard white spectralon target assuming reflectance fixed at 0.99 was used as a reference against the target objects. Thus, the reflectance values became dimensionless.

Fresh weight and dry weight for leaves, stems, and fruits for one standard maize plant per experimental plot as well as 3 to 4 standard plants for the field control were measured. The area-coefficient method was used to measure leaf area index (LAI) [14]. The same standard plant was used for biomass measurements. After the spectral measurements, gravimetric soil moisture (*θ*_m_, %) was measured by oven-drying soil samples in 2013 and 2014 while the volumetric soil moisture (*θ*_v_) for every 10 cm soil layer and 0–100 cm soil profiles were measured using the Diviner 2000 (Sentek Pty. Ltd., South Australia) in 2015. In 2013 and 2014, RSWC (%) was the ratio of *θ*_m_ and field capacity (*F*_c_): RSWC = (*θ*_m_/*F*_c_) × 100%. In 2015, RSWC = [*θ*_v_/(*F*_c_·BD)] × 100%, where BD is bulk density. Observation dates of biomass, LAI, and soil water content were the same as the spectral reflectance measurements.

### Water content indicators

Crop water indicators are calculated by leaf fresh weight (FW, g m^−2^), dry weight (DW, g m^−2^), and leaf area index (LAI) datasets. In 2013, 72 observations were conducted, 64 observations in 2014, 50 observations in 2015, and 186 datasets in total.

Canopy water content (CWC, g m^−2^), defined as the quantity of water per unit area of ground surface [26], is obtained by measuring product of the quantity of water per unit leaf area in g cm^−2^ and LAI [25], or calculated by the difference of FW and DW. In this study, CWC is the quantity of leaf water content in maize per unit area of ground surface calculated by Eq. (1) [27, 28].

1 $${\text{CWC}} = {\text{FW}}{-}{\text{DW}}$$

Leaf equivalent water thickness (EWT, g cm^−2^) at the leaf level usually equals the leaf water content per unit leaf area [25]. Here, at the canopy level, EWT is defined as the ratio between the quantity of water and the area, otherwise known as crop water content per unit leaf area (Eq. (2)) [25].

2 $${\text{EWT}} = \left( {{\text{FW}}{-}{\text{DW}}} \right)/{\text{LAI}}$$

Live fuel moisture content (LFMC, %) is the ratio of water mass to dry mass contained in live plant material. LFMC is determined by leaf moisture status, and closely correlated with seasonal changes of dry mass, which represents the quantity of available fuel [30, 36]. LFMC is calculated by Eq. (3) [41]:

3 $${\text{LFMC}} = \left[ {\left( {{\text{FW}}{-}{\text{DW}}} \right)/{\text{DW}}} \right] \times 100 = {\text{EWT}}/{\text{DMC}} \times 100$$

where, dry matter content (DMC, g cm^−2^) = DW/LAI.

### Spectral vegetation indices

In this study, we utilized four spectral vegetation indices (VIs), which are indirectly related with canopy water, NDVI, NR_red edge_, CI_green_, and CI_red edge_, and six water-sensitive spectral VIs including WI, MSI, SRWI, NDWI, NDWI_1640_, and NDWI_2130_ (Table 2). The goal of this study was to examine the spectral VIs’ potentials for estimating CWC, EWT, and LFMC.

**Table 2:** Vegetation indices (VIs) used in the study and related source references

Index	Formula	References
Indirect water-sensitive spectral VIs		
Normalized difference vegetation index (NDVI)	(*R*_*nir*_ − *R*_*red*_)/(*R*_*nir*_+ *R*_*red*_)	[42]
Red edge normalized ratio (NR_red edge_)	(*R*_750_ − *R*_710_)/(*R*_750_ + *R*_710_)	[43]
Green chlorophyll index (CI_green_)	(*R*_750_/*R*_550_) − 1	[44]
Red edge chlorophyll index (CI_red edge_)	(*R*_750_/*R*_710_) − 1	[44]
Direct water-sensitive spetral VIs		
Water index (WI)	*R*_900_/*R*_970_	[45]
Moisture stress index (MSI)	*R*_1600_/*R*_820_	[46]
Simple ratio water index (SRWI)	*R*_860_/*R*_1240_	[47]
Normalized difference water index (NDWI)	(*R*_860_ − *R*_1240_)/(*R*_860_ + *R*_1240_)	[48]
Normalized difference water index centered at 1640 nm (NDWI_1640_)	(*R*_858_ − *R*_1640_)/(*R*_858_ + *R*_1640_)	[49]
Normalized difference water index centered at 2130 nm (NDWI_2130_)	(*R*_858_ − *R*_2130_)/(*R*_858_ + *R*_2130_)	[49]

*R*_nir_ and *R*_red_ are the averaged reflectance among the waveband range to match MODIS data in the near-infrared (841–876 nm) and red (620–670 nm) wavelengths, respectively

### Data analysis

We used one-way ANOVA to analyze differences between crop water indicators, CWC, EWT, LFMC, and ten spectral VIs, NDVI, NR_red edge_, CI_green_, CI_red edge_, WI, MSI, SRWI, NDWI, NDWI_1640_, and NDWI_2130_, among treatment plots and control plots and field control. The relationships between ten spectral VIs and CWC, EWT, LFMC were also analyzed, respectively. All statistical analyses were performed with SPSS 17.0 software (SPSS, Chicago, IL, USA), and SigmaPlot 10.0 software (Systat, San Jose, CA, USA) was used to draw the figures.

## Results

### Responses of CWC, EWT, and LFMC to water stress

**Fig. 1:**
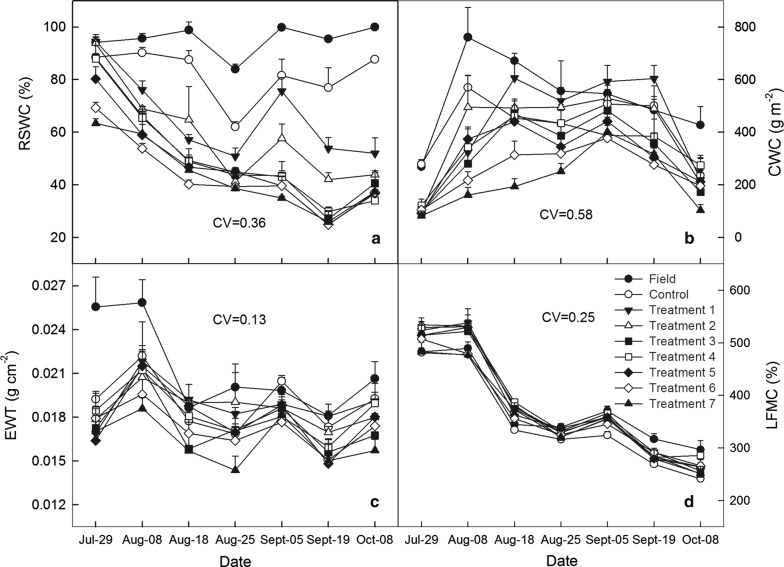
Variations in **a** relative soil water content *(*RSWC, %), **b** canopy water content (CWC, g m^−2^), **c** leaf equivalent water thickness (EWT, g cm^−2^), and **d** live fuel moisture content (LFMC, g cm^−2^) under different water treatments during the growing season of 2013 for maize agroecosystem. Treatments 1–7 indicate seven different irrigation amounts, 120 + 80, 100 + 40, 80, 60, 40, 25, and 15 mm as well as a rainfed control plots with no irrigation (Control) and a rainfed field control (Field)

Taking the data from the experiment in 2013 as an example, CWC, EWT, LFMC, and RSWC in response to different water treatments and their seasonal variation characteristics are shown in Fig. 1. The RSWC began to show clear changes from day 5 (29 July) after water irrigation controlling. Except for differences (*p* < 0.05) between field control and control plots as well as treatments 1 and 2 in the late periods in RSWC, differences (*p* < 0.05) among water treatments would gradually diminish with crop growth and development processes progressing, and ultimately approach (Fig. 1a). On 26 August, rewetting treatments with 40 and 80 mm made only for treatments 1 and 2 induced a slight increase of RSWC. Overall, differences (*p* < 0.05) in RSWC under different water treatments were the easiest to observe.

Among three crop water indicators, variations in CWC influenced by RSWC were the most prominent. CWC greatly differed among various irrigation levels and showed an obvious single-peak seasonal trend with a higher coefficient of variation (CV) value of 0.58. A maximum CWC value of 761.41 g m^−2^ was recorded on 8 August in well-watered plots while the peak values of CWC in drought-treated plots occurred later and a maximum lagging time might be about 1 month (Fig. 1b). EWT remained at a relatively stable level over the growth season with a lower CV value of 0.13 and a minimum peak value of 0.026 g cm^−2^, which occurred consistently with CWC (Fig. 1c). Differences (*p* < 0.05) in EWT between treatments and controls were not notable except for differences (*p* < 0.05) between field control and all other experimental plots at the initial stage of water controlling, which were no longer clear. Although there existed some fluctuations, EWT was still relatively consistent. Thus, EWT was not a valid indicator of maize water stress. Finally, LFMC showed a clearly decreasing trend over the whole season with an abrupt drop in mid-August and, after that, a weak peak value occurring around 5 September (Fig. 1d). LFMC had no remarkable treatment differences, which meant that water treatments had no effects on LFMC variation. Similarly, this made it difficult to monitor maize water stress.

### Responses of spectral VIs to water stress

**Fig. 2:**
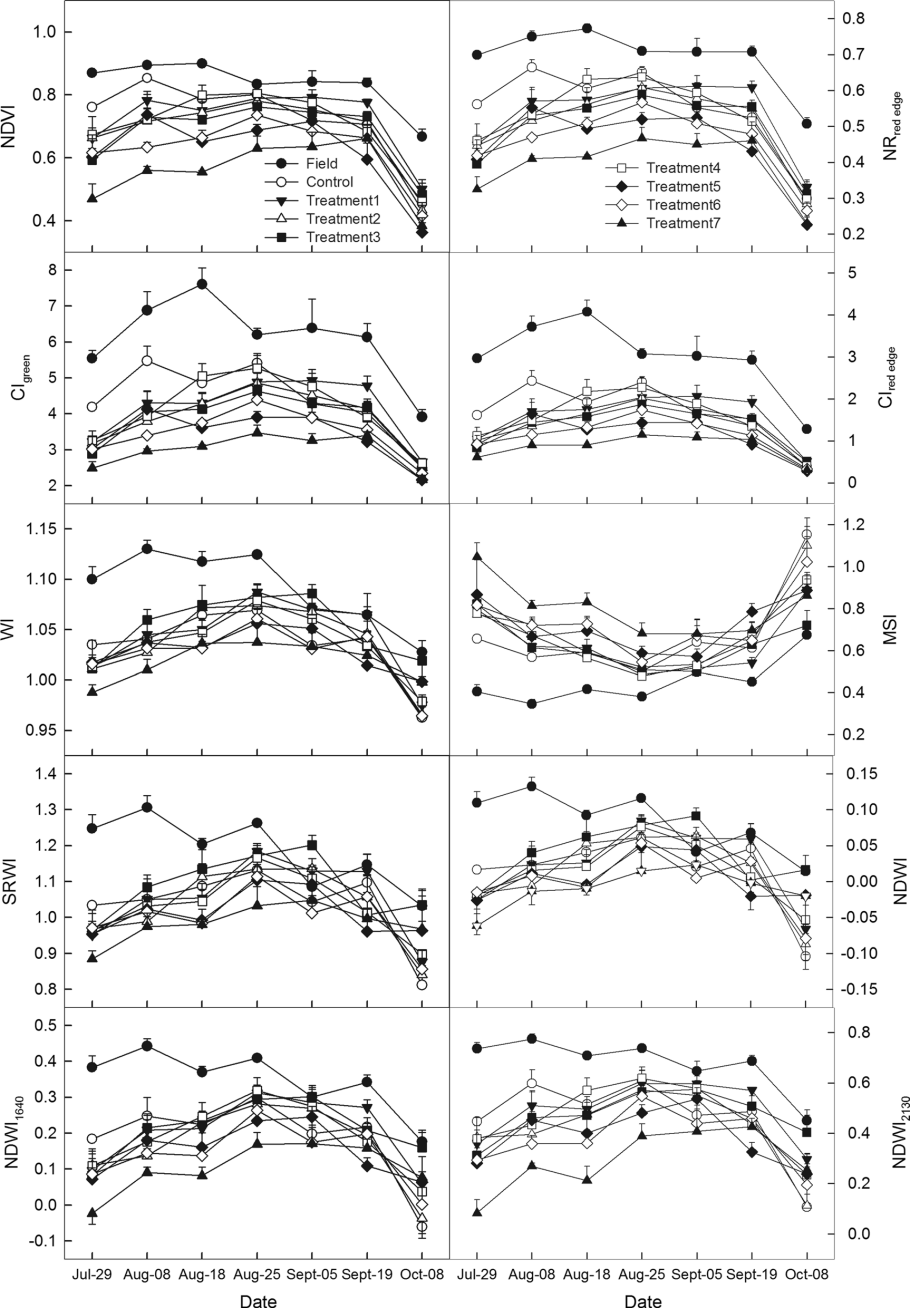
Variations in four indirect spectral vegetation indices, normalized difference vegetation index (NDVI), red edge normalized ratio (NR_red edge_), green chlorophyll index (CI_green_), red edge chlorophyll index (CI_red edge_), and six water-sensitive spectral vegetation indices, water index (WI), moisture stress index (MSI), simple ratio water index (SRWI), normalized difference water index (NDWI), normalized difference water index centered at 1640 nm (NDWI_1640_), and normalized difference water index centered at 2130 nm (NDWI_2130_) under different water treatments during the growing season of 2013 for maize agroecosystem. Treatments 1–7 indicate seven different irrigation amounts, 120 + 80, 100 + 40, 80, 60, 40, 25, and 15 mm as well as a rainfed control plots with no irrigation (Control) and a rainfed field control (Field)

Taking the observation datasets in 2013 as an example, Fig. 2 illustrates the effects of different water treatments on four indirect spectral VIs (NDVI, NR_red edge_, CI_green_, and CI_red edge_) and six direct water-sensitive spectral VIs (WI, MSI, RSWI, NDWI, NDWI_1640_, and NDWI_2130_), respectively. Except for MSI, other nine VIs to different degrees exhibited a single-peak seasonal trend (Fig. 2). If water conditions were drier, these nine VIs values would be relatively lower while MSI was higher, and the magnitudes of VIs increasing (or decreasing) during the whole season would be lower as well as the single peak seasonal trend would be weakened. Compared to crop water indicators (CWC, EWT, and LFMC), differences of VIs in response to different water treatments were more sensitive, especially NR_red edge_, CI_green_, and CI_red edge_ as well as NDWI_1640_ and NDWI_2130_. During the crop vegetative and reproductive stages, differences (*p* < 0.05) in crop growth recognized by NR_red edge_, CI_green_, and CI_red edge_ were stronger than NDWI_1640_ and NDWI_2130_.

During the initial stage of irrigation control, although the differences between water treatment plots were slight, ten VIs could recognize them between field control with control plots and treatment plots. With crop growth processes progressing, the differences were gradually amplified. During the peak growing season, such as on 8 August or 18 August, the effects of different water treatments on VIs were the most prominent. When crop entered into the reproductive stage, differences among every gradient plots recognized by VIs decreased. At the beginning of October (i.e., during the end of growing period), most VIs continued to decrease and there were no significant differences (*p* < 0.05) among control and treatment plots except for field control, or differences were no longer related to the irrigation treatments. Overall, among the ten spectral VI including six water-sensitive VIs (WI, MSI, RSWI, NDWI, NDWI_1640_, and NDWI_2130_), indirect spectral VIs (NR_red edge_, CI_green_, and CI_red edge_) were still the most sensitive to different water treatments.

### Estimations of crop water indicators by spectral VIs

**Fig. 3:**
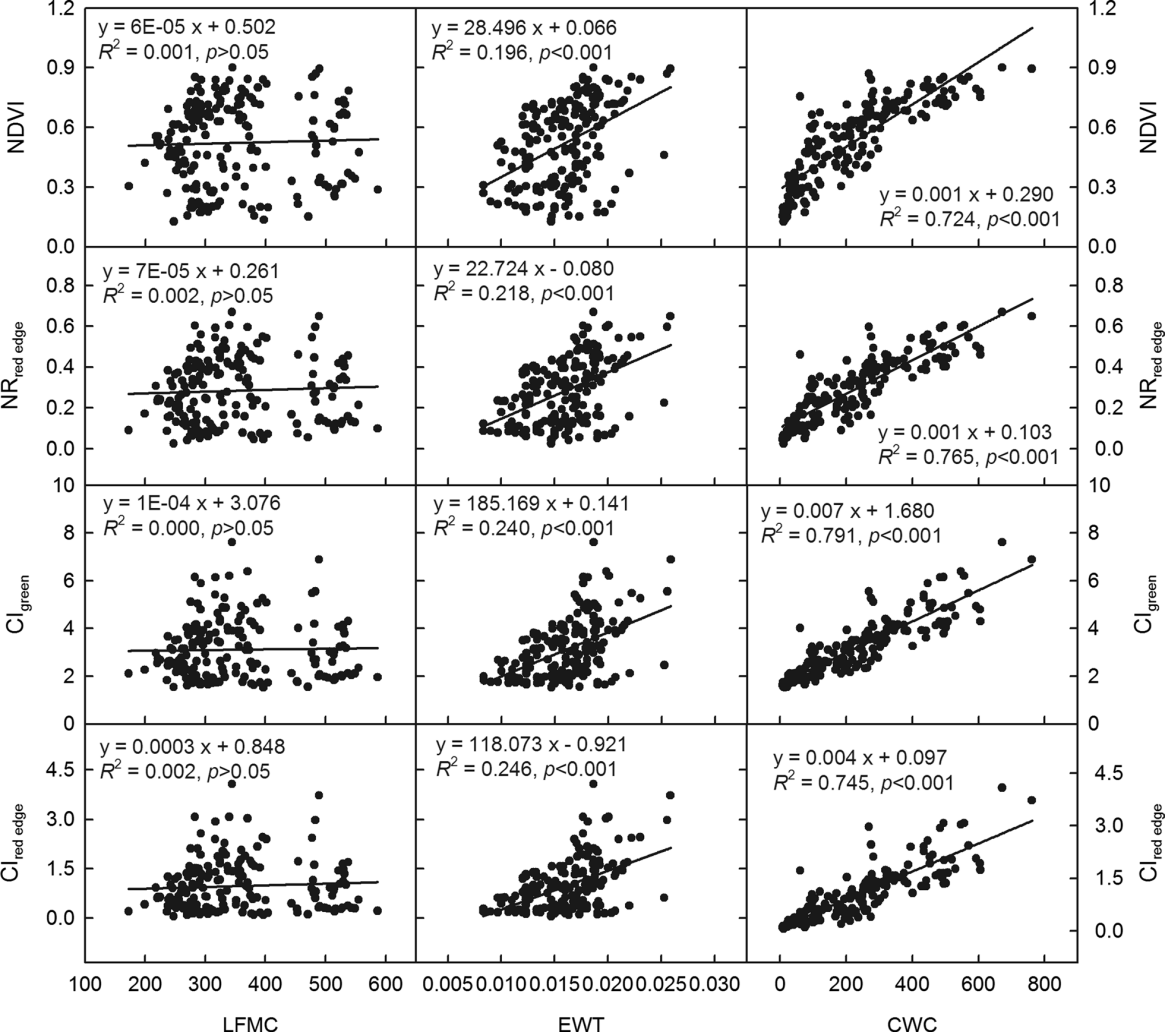
Correlation analysis of hyperspectral vegetation indices, normalized difference vegetation index (NDVI), red edge normalized ratio (NR_red edge_), green chlorophyll index (CI_green_), and red edge chlorophyll index (CI_red edge_) vs. canopy water content (CWC, g m^−2^), leaf equivalent water thickness (EWT, g cm^−2^), and live fuel moisture content (LFMC, g cm^−2^) for maize during the growing seasons from 2013 to 2015

**Fig. 4:**
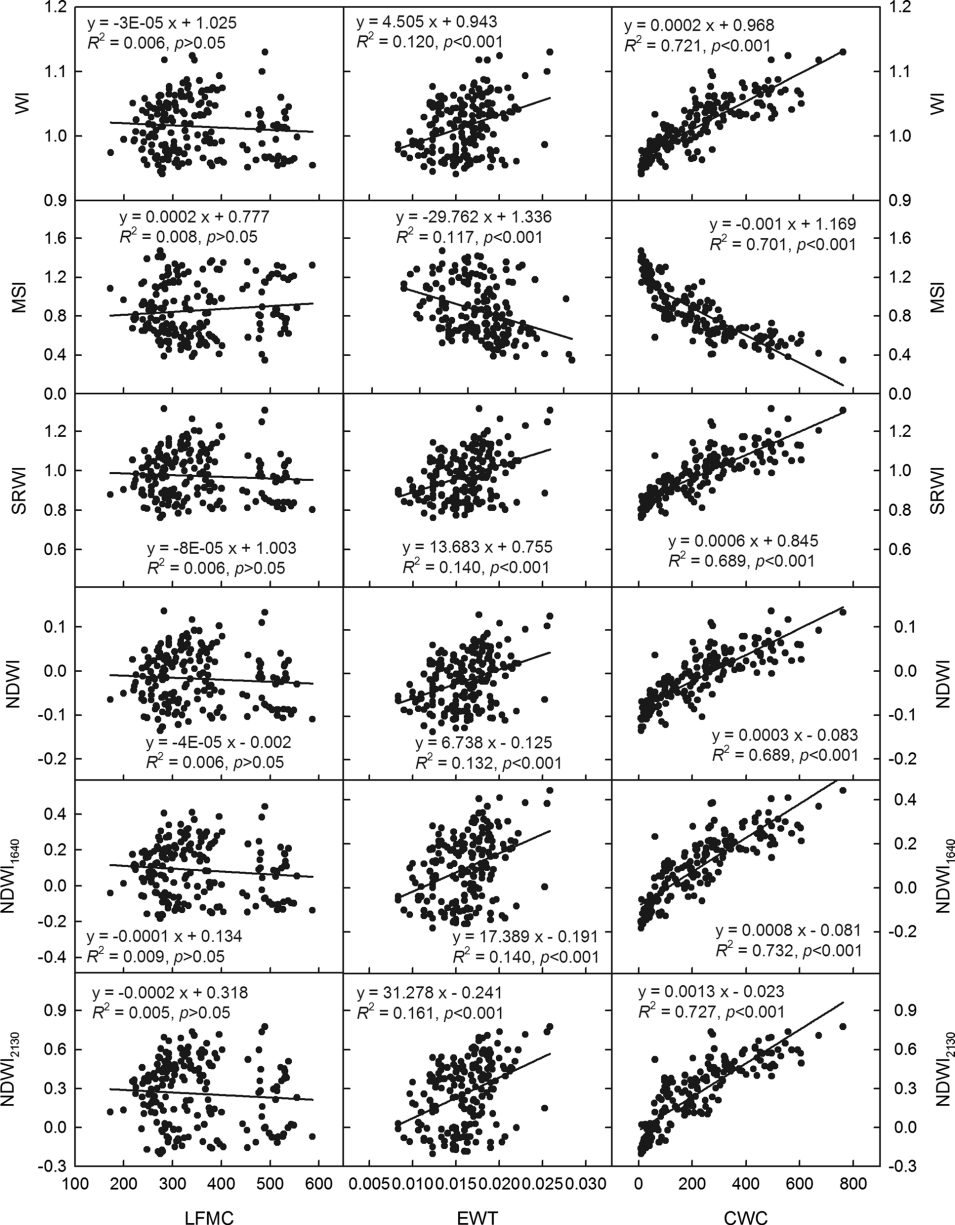
Correlation analysis of spectral vegetation indices, water index (WI), moisture stress index (MSI), simple ratio water index (SRWI), normalized difference water index (NDWI), normalized difference water index centered at 1640 nm (NDWI_1640_), and normalized difference water index centered at 2130 nm (NDWI_2130_) vs. canopy water content (CWC, g m^−2^), leaf equivalent water thickness (EWT, g cm^−2^), and live fuel moisture content (LFMC, g cm^−2^) for maize during the growing seasons from 2013 to 2015

Ten VIs including indirect spectral VIs (NDVI, NR_red edge_, CI_green_, and CI_red edge_) and direct water-sensitive spectral VIs (WI, MSI, SRWI, NDWI, NDWI_1640_, and NDWI_2130_) were used to estimate crop water indicators, LFMC, EWT, and CWC, separately (Figs. 3 and 4). Results showed that CI_green_, NR_red edge_, and CI_red edge_ in four indirect VIs showed better correlated relationships with CWC (*R*^2^ = 0.745–0.791, *p* < 0.001) and EWT (*R*^2^ = 0.218–0.246, *p* < 0.001) than NDVI (Fig. 3); While among six direct water-sensitive spectral VIs, NDWI_1640_ and NDWI_2130_ presented the highest sensitivity to CWC (*R*^2^ = 0.727–0.732, *p* < 0.001) and EWT (*R*^2^ = 0.140–0.161, *p* < 0.001) (Fig. 4). Overall, indirect spectral VIs, CI_green_, NR_red edge_, and CI_red edge_, which are closely related with crop growth, presented better prediction of crop water content than other six water-sensitive spectral VIs. Results also showed that LFMC obtained the poorest estimation and EWT was moderately estimated, while CWC had the best predictive power of water status (Figs. 3 and 4). So, compared to EWT and LFMC, CWC is the most ideal crop water indicator for monitoring crop water stress using field spectroscopy techniques.

## Discussion

### Responses of different crop growth stages to water variation

At different stages of crop growth and development, crop water demand is different [10, 50]. Moreover, the relationships between spectral water indices and plant water traitors were greatly affected by water stress, plant species, growing conditions and phenological stages [23, 51]. During crop growth and development processes, self-regulatory mechanisms exist for the crop itself [10, 52]. As such, more sensitivities of crop to water stress are reflected on crop growth and development rather than only on crop leaf structure or water status [15]. Many aspects of plant physiological processes are directly associated with plant tissue water instead of soil water supply capacity, and RSWC is evidently an indirect variable for crop growth [24, 53]. In this study, different water stress treatments resulted in different crop growth and development. These differences not only included differences in leaf water information, but also implied variance in leaf structural properties (e.g., LAI).

### Responses of spectral VIs to water variation

Visible signs of the plants responding to water stress are commonly curling, shrinking, and de-coloring of leaves as well as opening up of plant canopy and more [34]. For short-time water stress, plants may use photo-protection strategies to prevent damage, i.e. photosynthesis processes to decrease while heat emission and chlorophyll fluorescence processes increase [54]. And then, longer-time water stress will cause damages to chlorophyll pigments and changes for the leaf absorbance and reflectance [34]. Studies show that VIs are generally prone to reflect vegetation growth status, which commonly somewhat cancel spectral water information [15]. Actually, studies also demonstrate that there are some water-sensitive VIs which are only sensitive to water information but not vegetation growth status [11]. In this study, ten spectral VIs including four indirect water-sensitive spectral VIs, which are closely related with crop growth, and six direct water-sensitive spectral VIs, were utilized to estimate LFMC, EWT, and CWC, respectively. We found that indirect water-sensitive spectral VIs (CI_green_, NR_red edge_, and CI_red edge_) showed better sensitivity to crop water indicators than any other water-sensitive spectral VIs. This study demonstrates that CWC considering crop growth and development information has the best predictive power of crop water status. Furthermore, this study illustrates that it will be very limited for accurately monitoring crop water status unless crop water indicators not only include water information but also contain biomass or LAI knowledge.

### Relationships of crop water indicators

CWC, EWT, LFMC, and RWC are different variables for describing vegetation water status [25, 27, 28, 32, 33, 35, 41]. LFMC is considered to an optimum indicator for detecting vegetation water information especially for fire danger assessment [33]. Nevertheless, it is difficult to directly estimate LFMC using optical remote sensing [4]. RWC could sufficiently reflect crop water stress, but it is also difficult to obtain leaf spectral information at turgor. Thereby, RWC could not be estimated directly utilizing optical remote sensing techniques. Many studies have been performed based on multi-species, multi-functional types, multi-regional or leaf dehydration experiments to remotely estimate EWT [23, 52, 55–57]. However, few studies have been conducted based on a single species with a wide range of plant water content spanning well-watered to water-stressed conditions. In this study, a wide range of water content with the EWT value of 0.014 g cm^−2^–0.026 g cm^−2^ in 2013 and the lowest value reaching 0.008 g cm^−2^ after 3 years of datasets (2013–1015) were considered, which has not been reported before. This study includes not only extreme drought values under water-stressed conditions, but also extreme moist values under well-watered conditions.

Moreover, LFMC is relatively stable for a single species. As such, it is not suitable for detecting water stress status for the same species. Similarly, less EWT variation was observed and EWT also stayed relatively stable over the whole season in this study. To maintain a level compatible with its basic functions, leaf water content per unit leaf area actually does not vary much due to moderate water stress, at the same time, leaf structure and dry mass also affects remote estimation of leaf water content [52]. Although both LFMC and EWT could sufficiently reflect crop water content when photosynthesis occurs under the condition of water stress, they are stress-adapted state variables responding to water stress and could not accurately describe the accumulated effects of water stress.

However, greatly differing from EWT and LFMC, CWC not only includes canopy water content information, but also is closely correlated with LAI, which means that CWC itself not only contains crop growth and development information reflecting crop water content, but also maintains accumulation effects of water stress [15]. Furthermore, CWC to some degrees could reflect an instantaneous status of crop water at a particular moment. Studies showed that LAI is essential for assessing vegetation water status [33]. In addition, de Jong et al. [34] also found that LAI could be important for estimating leaf water content using hyperspectral remote sensing. Therefore, for the same kind of crop, CWC is an important water content parameter at canopy scale obtained by upscaling leaf water content via LAI, which can effectively present crop growth and water condition. This study confirms that CWC is an optimal indicator of crop water stress status and remote monitoring.

## Conclusions

In the present study, we compared the responses of CWC, EWT, and LFMC to water stress treatments and their estimations using ten spectral VIs based on canopy reflectance and its corresponding biological and environmental observation datasets in summer maize for 2013–2015 consecutively 3 years. The following conclusions can be drawn: (i) Compared to EWT and LFMC, CWC is more sensitive to different water treatments, and is a valid indicator of crop water stress. (ii) Indirect water-sensitive spectral VIs, CI_green_, NR_red edge_, and CI_red edge_ were the best predictive VIs for CWC. (iii) CWC considering crop growth and development information had the best predictive power of crop water status using hyperspectral VIs. (iv) CWC is a comprehensive indicator reflecting the health and vigor of crop growth, thus, CWC is the most promising for indicating crop water content and monitoring crop water stress using field spectroscopy techniques. In future study, consideration of the inherent mechanism of crop water stress as well as crop morphological and structural properties, coupled with hyperspectral methods, will be used to monitor crop water status. In addition, this study was conducted only based on a single site and crop and consecutively limited 3 years of datasets, thus, studies on multi-species, larger regions, and longer periods should be assessed, which is of significant importance in determining useful information for drought assessment and agriculture decisions regarding irrigation in order to reduce the effects of drought on crop growth.

## Abbreviations

BD: bulk density
CI_green_: green chlorophyll index
CI_red edge_: red-edge chlorophyll index
CWC: canopy water content
DMC: dry matter content
DW: dry weight
EWT: leaf equivalent water thickness
*F*_c_: field capacity
FW: fresh weight
*θ*_m_: gravimetric soil moisture
LAI: leaf area index
LFMC: live fuel moisture content
MSI: moisture stress index
NDVI: normalized difference vegetation index
NDWI: normalized difference water index
NDWI_1640_: normalized difference water index centered at 1640 nm
NDWI_2130_: normalized difference water index centered at 2130 nm
NR_red edge_: red edge normalized ratio
RSWC: relative soil water content
RWC: relative water content
SRWI: simple ratio water index
VIs: vegetation indices
*θ*_v_: volumetric soil moisture
WI: water index
